# The relationship between mixed venous blood oxygen saturation and pulmonary arterial and venous pressures in patients with heart failure

**DOI:** 10.14814/phy2.70128

**Published:** 2024-11-20

**Authors:** Ryuji Funaki, Kazuo Ogawa, Yuto Mashitani, Takuya Oh, Yusuke Kashiwagi, Toshikazu D. Tanaka, Tomohisa Nagoshi, Makoto Kawai, Michihiro Yoshimura

**Affiliations:** ^1^ Division of Cardiology, Department of Internal Medicine The Jikei University School of Medicine Tokyo Japan

**Keywords:** heart failure, intrapulmonary bronchopulmonary anastomose, mixed venous blood oxygen saturation, pulmonary arterial pressure, pulmonary venous pressure

## Abstract

Recent discoveries have identified intrapulmonary bronchopulmonary anastomoses (IBAs) as a relatively common phenomenon forming intrapulmonary right‐to‐left shunts. This study hypothesizes that IBAs play a significant role in the pathophysiology of heart failure. We aim to investigate the impact of these intrapulmonary right‐to‐left shunts on pulmonary arterial and venous pressures in heart failure patients, utilizing mixed venous oxygen saturation (SvO₂) as a key measurement. This study included 237 patients with heart failure who underwent cardiac catheterization. The relationships between SvO₂ and pulmonary artery systolic pressure (sPAP), pulmonary artery wedge pressure (PAWP), and left ventricular end‐diastolic pressure (LVEDP) were examined using various statistical methods (single regression analysis, partial correlation analysis, structural equation modeling, and Bayesian estimation). All statistical methods that we performed showed that SvO₂ was significantly and negatively correlated with both sPAP and PAWP (*p* < 0.01, respectively). However, SvO₂ did not significantly correlate with LVEDP. These results suggest that a decrease in SvO₂ leads to an increase in PAWP and sPAP, while LVEDP is only passively influenced by PAWP. This phenomenon likely reflects the impact of an intrapulmonary right‐to‐left shunt caused by IBAs. The decrease in SvO₂ causes an increase in sPAP and may also cause an increase in PAWP via IBAs.

## 1 INTRODUCTION

The pulmonary artery is primarily known for its involvement in gas exchange within the lungs. However, under certain conditions, it can also contribute to the nutrient supply to lung tissue along with the bronchial arteries, which can be observed clinically. For example, in the treatment of hemoptysis, the bronchial artery may be occluded, and in such cases, the pulmonary artery supplies the necessary nutrient blood to the lungs (Hwang et al., 2021). Furthermore, in lung transplantation, the bronchial artery is not always anastomosed, and in such cases, the pulmonary artery plays a compensatory role in nourishing the lungs (Tong et al., 2015).

Research is currently being actively conducted on the anastomosis between the pulmonary artery and the bronchial artery within the lungs. These anastomoses are called “intrapulmonary bronchial artery anastomoses (IBAs).” These anastomoses connect the pulmonary artery and the bronchial artery, bypassing the alveoli. IBAs are prominent during the fetal period and usually close at birth, but they persist in diseased states (McMullan et al., 2004). As a result, in various lung diseases, a right‐to‐left shunt can occur, potentially causing severe hypoxia (Bush et al., 2017; Dorfmüller et al., 2014; Galambos et al., 2014). These anastomoses, ranging in diameter from 15 μm to 500 μm, redistribute non‐oxygenated blood, causing blood flow insufficiency in the peripheral sites of the lungs (Lovering et al., 2010). These shunt vessels can cause hypoxia during exercise, hypoxic conditions, and catecholamine release, but they are also seen in healthy individuals (Eldridge et al., 2004; Laurie et al., 2012; Lovering et al., 2006). Notably, these vascular connections are prominent in the lungs affected by COVID‐19, reflecting the mobilization of shunt vessels (Galambos et al., 2021). This phenomenon explains the increased likelihood of hypoxia in COVID‐19 patients, causing right‐to‐left shunts within the lungs. Therefore, IBAs may play a more significant role in various conditions and diseases than previously thought. However, while IBAs are anatomically present, their hypothesized functionality has not been consistently elucidated. Thus, rigorous validation of their functionality using diverse methodologies across various pathological conditions is essential. To date, the relationship between IBAs and heart failure has not been studied. We aim to investigate the hemodynamic impact of intrapulmonary shunts possibly caused by IBAs.

Mixed venous oxygen saturation (SvO₂) is a parameter measured using pulmonary arterial blood, where blood from the superior and inferior vena cava, as well as the coronary sinus, is mixed. It reflects the amount of oxygen remaining in the venous blood and potentially indicates the balance between systemic oxygen supply and demand. Clinically, SvO₂ is a critical marker for systemic management because it reflects both respiratory and cardiovascular function. However, despite its significance in guiding treatment strategies, there has been limited clinical research and insufficient discussion on SvO₂, primarily because it requires invasive sampling from the pulmonary artery. Herein, we believe that SvO₂ is a crucial indicator of oxygenation in the blood flowing toward the lungs. A decrease in SvO₂ could lead to increased pulmonary artery pressure and, if there is an intrapulmonary righ‐to‐lefet shunt, increased pulmonary venous pressure.

In the lungs of heart failure patients, we believe that intrapulmonary right‐to‐left shunts occur to some extent via IBAs. Hypoxia in the pulmonary arteries is thought to not only cause pulmonary hypertension but also pulmonary venous hypertension. This study aims to investigate the relationship between SvO₂, an indicator of hypoxia in the pulmonary artery, and systolic pulmonary artery pressure (sPAP), pulmonary artery wedge pressure (PAWP) as an indicator of pulmonary venous pressure, and left ventricular end‐diastolic pressure (LVEDP).

## 2 MATERIALS AND METHODS

### 2.1 Study participants

The study population consisted of 237 heart failure patients who underwent right cardiac catheterization between June 2017 and May 2022. Patients who were administered oxygen or who had obvious pulmonary arterial hypertension were excluded. Pulmonary arterial hypertension was defined as a mean pulmonary arterial pressure of 20 mmHg or greater and PAWP of 15 mmHg or less, according to the latest findings on pulmonary hypertension (Simonneau et al., 2019). We excluded patients with right and left shunts such as those with atrial and/or ventricular septal defects.

### 2.2 Ethics

This study was approved by the Ethics Committee of The Jikei University School of Medicine for Biomedical Research (study protocol: 24‐355(7121)). We complied with the routine ethical regulations of our institution. All clinical investigations were conducted in accordance with the principles set forth in the Declaration of Helsinki. As this was a retrospective study, instead of obtaining informed consent from each patient, we posted a notice about the study design and contact information according to our routine ethical regulations on the official website of our institution (https://jikei.bvits.com/rinri/publish.aspx). In this public notification, we ensured that patients had the opportunity to refuse to participate (opt‐out) in the study.

### 2.3 Performance of right and left cardiac catheterizations

Right heart catheterization was performed using a thermodilution catheter (Bioptimal Japan, Tokyo, Japan) as a Swan‐Ganz catheter. Blood for measuring SvO₂ was drawn with the distal end of the thermodilution catheter, which was in the main pulmonary artery. The position of the catheter was confirmed using a pressure waveform and fluoroscopy. After discarding the first 10 mL of blood, the rest was collected using a syringe for blood gas analysis, and the residual air was quickly removed. Blood gas samples were immediately analyzed using a blood gas analyzer (ABL800 FLEX; RADIOMETER, Denmark) to measure SvO₂.

Left heart catheterization was performed using a 4 or 5 Fr Judkins right catheter (Terumo, Tokyo, Japan). The catheter position was confirmed using a pressure waveform and fluoroscopy, and measurements were performed at the center of the left ventricular cavity.

sPAP and PAWP were measured generally within 10 min after measuring SvO₂. LVEDP was measured generally within 30 min after measuring SvO₂.

### 2.4 Blood collection data

Blood data were collected by drawing blood at the start of right heart catheterization. A Radifocus Introducer IIH (Terumo, Tokyo, Japan) was placed in the femoral or internal jugular vein, the first 10 mL of blood was discarded, and the sample was collected using a 10 mL piston. The tests were performed in a laboratory.

### 2.5 Statistical analysis

Statistical analyses were performed using the IBM SPSS software (version 27; SPSS Inc., Chicago, IL, USA). Continuous variables are expressed as mean ± standard deviation (SD) and median (upper and lower quartiles). The analyses included single regression and partial correlation. Statistical significance was set at *p* < 0.05.

Structural equation modeling and Bayesian estimation were performed using the IBM SPSS Amos (version 27; Amos Development Corporation, Meadville, PA, USA). For this purpose, we used our previous studies as references (Hasegawa et al., 2021; Suzuki et al., 2021; Uno et al., 2019; Yamada et al., 2020). The marginal posterior distributions of the bivariate correlations obtained from the Bayesian estimation results are represented as frequency distribution polygons plotted by two‐dimensional contour lines, with black representing 95%; dark gray, 90%; and light gray, 50% confidence intervals.

Based on previous reports, SvO₂ is known to be associated with arterial blood oxygen saturation, oxygen consumption, cardiac output (CO), and hemoglobin (Hb) (Kotake et al., 2009). Therefore, it is important to consider the possibility that the relationships between SvO₂ and sPAP, PAWP, and LVEDP could be influenced by these factors. To address this, partial correlation analysis was conducted with respect to cardiac index (CI) and Hb for which data could be obtained in our database.

## 3 RESULTS

### 3.1 Clinical characteristics of patients

The clinical characteristics of the patients are summarized in Table 1. Mean SvO₂, sPAP, PAWP, and LVEDP were 67.5 ± 7.5%, 26.4 ± 10.5 mmHg, 11.8 ± 7.7 mmHg, and 14.0 ± 7.1 mmHg, respectively.

**TABLE 1:** Clinical characteristics of patients.

	Mean ± SD or *N* (%), median (interquartile range)
Number of patients	237
Age (year)	68.1 ± 13.6, 70.0 (60.0–79.0)
Sex (male) (%)	178 (75.1%)
Body mass index (kg/m^2^)	23.2 ± 5.1, 22.7 (20.3–25.6)
SvO₂ (%)	67.5 ± 7.5, 68.0 (63.8–72.9)
LVEF (%)	43.6 ± 15.2, 42.3 (32.4–54.0)
SVI (mL/m^2^)	37.6 ± 11.7, 37.1 (30.1–43.7)
CI (L/min/m^2^)	2.6 ± 0.7, 2.5 (2.1–3.0)
sPAP (mmHg)	26.4 ± 10.5, 24.0 (19.0–31.0)
PAWP (mmHg)	11.8 ± 7.7, 10.0 (6.0–17.0)
LVEDP (mmHg)	14.0 ± 7.1, 13.0 (9.0–19.0)
WBC (1000/μL)	6.8 ± 3.3, 6.1 (5.0–7.6)
Hb (g/dL)	12.8 ± 2.3, 12.9 (11.3–14.5)
Cr (mg/dL)	2.3 ± 3.0, 1.1 (0.9–1.6)
eGFR (mL/min/m^2^)	47.6 ± 27.4, 51.0 (28.0–65.6)
UA (mg/dL)	6.6 ± 2.1, 6.5 (5.1–7.9)
Na (mmol/L)	139.1 ± 3.3, 139.0 (137.0–141.0)
K (mmol/L)	4.3 ± 0.5, 4.3 (4.0–4.6)
FBS (mg/dL)	114.7 ± 40.1, 102.0 (90.0–129.0)
HbA1c (%)	6.2 ± 1.0, 5.9 (5.6–6.5)
TG (mg/dL)	106.1 ± 46.0, 95.5 (72.0–130.0)
HDL‐C (mg/dL)	50.3 ± 15.5, 48.0 (39.3–59.8)
LDL‐C (mg/dL)	100.7 ± 32.5, 96.5 (74.0–125.5)
LDL‐C/HDL‐C	2.1 ± 0.8, 2.0 (1.5–2.7)
MDA‐LDL (U/L)	101.1 ± 37.6, 96.0 (75.8–121.0)
Alb (g/dL)	3.5 ± 0.5, 3.6 (3.2–3.9)
CRP (mg/dL)	1.1 ± 2.4, 0.3 (0.1–0.9)
BNP (pg/mL)	399.1 ± 507.7, 214.6 (102.9–461.5)
Medication	
Diuretics (%)	134 (56.5%)
ACE inhibitors/ARBs (%)	142 (59.9%)
MRAs (%)	92 (38.8%)
ARNIs (%)	21 (8.9%)
SGLT2 inhibitors (%)	17 (7.2%)
Beta‐blockers (%)	165 (69.6%)
CCBs (%)	77 (32.5%)

Abbreviations: ACE, angiotensin‐converting enzyme; Alb, albumin; ARBs, angiotensin II receptor blockers; ARNIs, angiotensin receptor neprilysin inhibitors; BNP, brain natriuretic peptide; CCBs, calcium channel blockers; CI, cardiac index; Cr, creatinine; CRP, C‐reactive protein; eGFR, estimated glomerular filtration rate; FBS, fasting blood sugar; Hb, hemoglobin; HbA1c, hemoglobin A1c; HDL‐C, high density lipoprotein; K, potassium; LDL‐C, low density lipoprotein; LVEDP, left ventricular end‐diastolic pressure; LVEF, left ventricular ejection fraction; MAD‐LDL, malondialdehyde‐modified low‐density lipoprotein; MRAs, mineralocorticoid receptor antagonists; Na, sodium; PAWP, pulmonary artery wedge pressure; SD, standard deviation; SGLT2, sodium‐glucose co‐transporter type 2; sPAP, pulmonary artery systolic pressure; SVI, stroke volume index; SvO₂, mixed venous blood oxygen saturation; TG, triglycerides; UA, uric acid; WBC, white blood cell count.

### 3.2 Relationship between SvO₂ and each cardiopulmonary pressure

**FIGURE 1:**
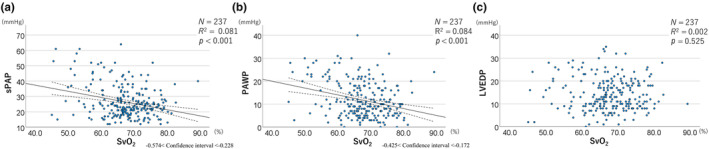
Regression analysis showing the correlation between SvO₂ and sPAP, PAWP, and LVEDP. A significant negative correlation is found between SvO₂ and sPAP (a) and PAWP (b), but not with LVEDP (c). About (a) and (b), the dotted curves that exist above and below the regression line represent 95% confidence intervals. LVEDP, left ventricular end‐diastolic pressure; PAWP, pulmonary artery wedge pressure; sPAP, pulmonary artery systolic pressure; SvO₂, mixed venous blood oxygen saturation.

To analyze the relationship of SvO₂ with sPAP, PAWP, and LVEDP, a single regression analysis was performed between each factor. As shown in Figure 1, SvO₂ showed a significant negative correlation with sPAP (*p* < 0.001, *R* ^2^ = 0.081, −0.574 < 95% confidence interval <−0.228) and PAWP (*p* < 0.001, *R* ^2^ = 0.084, −0.425 < 95% confidence interval <−0.174) but no significant relationship with LVEDP (*p* = 0.525).

### 3.3 Independence of the relationship between “SvO₂ and sPAP” and “SvO₂ and PAWP”

**FIGURE 2:**
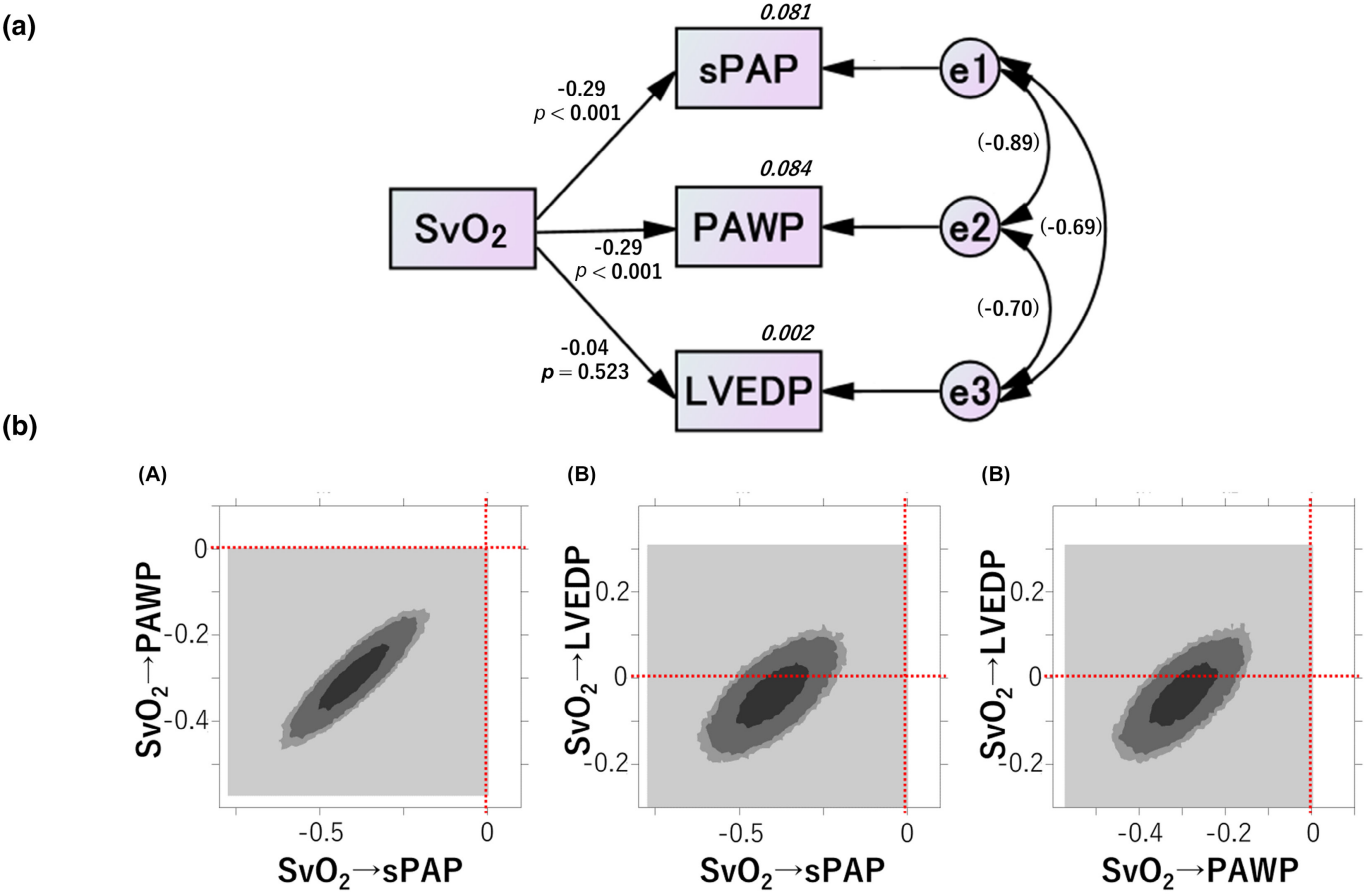
Structural equation modeling and Bayesian estimation. This path diagram in (a) shows the effect of SvO₂ on sPAP, PAWP, and LVEDP. Standardized coefficients, squared coefficients of multiple correlations (in italics), and correlation coefficients of the exogenous variables (in square brackets) are shown. SvO₂ is significantly correlated with sPAP and PAWP (*p* < 0.001, respectively), but not with LVEDP (*p* = 0.523). Additionally, e1, e2, and e3 are significantly correlated (*p* < 0.001). The results of Bayesian estimation in (b) using Amos Graphics are shown in a two‐dimensional contour image. From the center of the figure, the three colors are black, dark gray, and light gray, where black represents 95%; dark gray, 90%; and light gray, 50% confidence intervals. It is visually clear from this figure that the effects of SvO₂ on sPAP and PAWP are in the negative range, far from zero, strongly suggesting a negative effect on both (A); however, the effect of SvO₂ on LVEDP is on zero line, suggesting no effect on LVEDP (B, C). LVEDP, left ventricular end‐diastolic pressure; PAWP, pulmonary artery wedge pressure; sPAP, pulmonary artery systolic pressure; SvO₂, mixed venous blood oxygen saturation.

In addition to the above considerations, the independence of the relationship between “SvO₂ and sPAP”, “SvO₂ and PAWP”, and “SvO₂ and LVEDP” was examined using structural equation modeling. To evaluate the relationship between sPAP, PAWP, and LVEDP as closely as possible, a path diagram was devised, as shown in Figure 2a. The paths between the variables are represented by one‐way arrows from the independent to the dependent variable, representing positive or negative effects, and by two‐way arrows between the two variables, representing correlations. The dependent variable is accompanied by an error variable, the one‐way arrow by an estimate of the standardized coefficient, and the two‐way arrow by an estimate of the correlation coefficient. The results of this structural equation modeling are presented in Figure 2a, where SvO₂ was found to have a significant negative relationship with sPAP and PAWP (*p* < 0.001, respectively) but no significant relationship with LVEDP (*p* = 0.523). The standardized coefficients were SvO₂ to sPAP with an estimated value of −0.285, to PAWP with an estimated value of −0.290, and to LVEDP with an estimated value of −0.041.

Bayesian estimation was also performed for the difference in the involvement of SvO₂ on sPAP, PAWP, and LVEDP. As shown in Figure 2b, the distribution of the correlations between the two variables was visualized as a two‐dimensional contour image, and the effects of SvO₂ on sPAP and PAWP were in the negative range, far from zero, strongly suggesting a negative effect on both; however, the effect of SvO₂ on LVEDP was on the zero line, suggesting no effect on LVEDP.

The results of structural equation modeling and Bayesian estimation indicated that “SvO₂ and sPAP” and “SvO₂ and PAWP” had independent relationships with each other; however, “SvO₂ and LVEDP” had no relationship.

### 3.4 Examination of the possible involvement of CO and Hb

As aforementioned, SvO₂ could theoretically be affected by CO and Hb (Kotake et al., 2009). Therefore, we performed a partial regression analysis to verify that CI and Hb levels did not affect the key findings of the present study, the relationship between “SvO₂ and sPAP” and “SvO₂ and PAWP.” As shown in Table 2, the relationships between “SvO₂ and sPAP” and “SvO₂ and PAWP” were maintained when CI and Hb were included simultaneously in the control factors (*p* < 0.001, respectively).

**TABLE 2:** Partial correlation analysis showing the relationships between “SvO₂ and sPAP” and “SvO₂ and PAWP” with Hb and CI as control variables.

Control variable (1)	Control variable (2)	Independent variable	<−‐>	Independent variable	Partial correlation coefficient	P‐value
Hb	CI	SvO₂	<−‐>	sPAP	−0.292	<0.001
Hb	CI	SvO₂	<−‐>	PAWP	−0.284	<0.001

Abbreviations: CI, cardiac index; Hb, hemoglobin; PAWP, pulmonary artery wedge pressure; sPAP, pulmonary artery systolic pressure; SvO₂, mixed venous blood oxygen saturation.

### 3.5 Gender‐based analysis

We conducted the aforementioned analysis separately for males and females. The results of the structure equation modeling are presented below. For males, SvO₂ exhibited a significant negative correlation with sPAP and PAWP (*p* < 0.001, respectively), but no significant correlation with LVEDP (*p* = 0.801). For females, SvO₂ also demonstrated a significant negative correlation with sPAP and PAWP (*p* < 0.001, respectively), while no significant correlation was observed with LVEDP (*p* = 0.316). In summary, no significant differences were found between genders.

## 4 DISCUSSION

### 4.1 Intrapulmonary right‐to‐left shunt mechanism in heart failure: A hypothetical pathophysiology

Based on the previous study by Galambos, we explain our hypothesis (Galambos et al., 2014, 2021). In heart failure, especially with pulmonary congestion, the following pathophysiology is assumed to occur.

First, in a normal lung, deoxygenated blood in the pulmonary arteries enters the alveolar capillary bed for gas exchange, and oxygenated blood is collected via pulmonary veins and enters the left heart. A small amount of oxygenated blood supplies the terminal bronchiole via the bronchial artery and the bronchiolar capillary network. Deoxygenated blood is collected by the bronchial vein and subsequently enters the left heart via the pulmonary vein. In healthy individuals, IBAs are mostly closed, and there is minimal blood flow between the pulmonary and bronchial vascular trees.

In the lungs of patients with heart failure, pulmonary congestion impedes the deoxygenated blood in the distal pulmonary arteries from reaching the alveolar capillary network for gas exchange. Instead, it is redirected through open IBAs toward the bronchial arteries and bronchial microcirculation, thereby bypassing the alveolar capillary bed. Typically, arteriolar pressure is approximately 36 mmHg (Darwish & Lui, 2024). Although direct measurements of arterioles derived from bronchial arteries are not available, it can be surmised that such a low pressure would facilitate adequate blood inflow from the pulmonary artery to the bronchial arterioles and bronchial microcirculation via the IBAs. Furthermore, hypoxia‐induced elevation of pulmonary artery pressure may exacerbate this phenomenon. The bronchial arteries, capillaries, and veins are passively dilated due to the massive amount of blood coming from the right heart. The blood remains deoxygenated and is collected by the bronchial veins, entering the left heart via the pulmonary veins that anastomose with the bronchial veins. We hypothesize that this mechanism leads to the formation of intrapulmonary right‐to‐left shunts.

### 4.2 Relationship between hypoxia indicated by SvO₂ and sPAP

This study revealed a significant negative relationship between SvO₂ and sPAP. The main mechanism is hypoxic vasoconstriction, an intrinsic oxygenation homeostasis mechanism in the pulmonary vasculature (Dunham‐Snary et al., 2017). Furthermore, it suggests that persistent hypoxia enhances vasoconstriction and remodeling of the pulmonary vessels through the activation of hypoxia‐inducible factor and other factors, leading to persistent pulmonary hypertension.

### 4.3 Relationship between hypoxia indicated by SvO_2_ and PAWP

It is assumed that pulmonary venous hypertension occurs when the pulmonary veins are exposed to hypoxia, and as discussed below, the mechanisms underlying this have been well studied recently. Pulmonary veins have been shown to respond to several vasoconstrictors, including endothelin (Aharinejad et al., 1995; Raj et al., 1992; Toga et al., 1992), platelet‐activating factor (Toga et al., 1992), and thromboxane (Kadowitz & Hyman, 1980; Raj et al., 1990), and it has been shown that pulmonary veins contract even under hypoxia at the animal experimental level (Dingemans & Wagenvoort, 1978; Hillier et al., 1997; Raj et al., 1990; Raj & Chen, 1986; Sheehan et al., 1992; Tracey et al., 1989; Zhao et al., 1993). In humans, pulmonary vascular remodeling and thickening of the pulmonary venous intima have been observed in patients with pulmonary venous hypertension and heart failure (Fayyaz et al., 2018). Although the pulmonary veins contain fewer smooth muscle cells than the pulmonary arteries, they have been reported to be significantly altered in response to many stimuli (Arrigoni et al., 1999; Schindler et al., 2007).

### 4.4 Clinical significance of this study

This study demonstrated that in heart failure, a decrease in SvO₂ could lead to an increase in not only pulmonary arterial pressure but also pulmonary venous pressure. This suggests that a decrease in SvO₂ could be a cause of not only right heart failure but also left heart failure. Generally, as left heart failure becomes more severe, SvO₂ is expected to decrease, indicating the formation of a so‐called vicious cycle. Improving oxygenation is extremely important for the improvement of heart failure, and SvO₂ has been reaffirmed as a valuable indicator in this regard.

### 4.5 Study limitations

This study has several limitations. First, this study is a single‐arm study and does not include a control group. However, it is not feasible to insert a Swan‐Ganz catheter in healthy individuals or those with mild heart failure, so this limitation is unavoidable. In the future, it may be necessary to consider animal studies. Second, SvO₂ may be related to arterial oxygen saturation, oxygen consumption, CO, and Hb. In this study, CI and Hb levels were investigated, but arterial oxygen saturation and oxygen consumption could not be evaluated. This point needs to be studied more accurately. Finally, although we analyzed heart failure as a whole in this study, it is necessary to conduct similar analyses based on the underlying diseases of heart failure.

## 5 CONCLUSION

In patients with heart failure, a decrease in SvO₂ is associated with an increase in pulmonary venous pressure, independent of pulmonary artery pressure. Although it is a hypothesis, this phenomenon likely reflects the impact of an intrapulmonary right‐to‐left shunt caused by IBAs.

## AUTHOR CONTRIBUTIONS

R.F., K.O., and M.Y. conceived and designed research; R.F., Y.M., T.O., and Y.K. performed experiments; R.F., K.O., and T.D.T. analyzed data; R.F., K.O., T.N., M.K., and M.Y. interpreted results of experiments; R.F., K.O., Y.M., T.O., Y.K., and T.D.T. prepared figures; R.F., K.O., and M.Y. drafted manuscript; R.F., K.O., T.N., M.K., and M.Y. edited and revised manuscript; R.F., K.O., Y.M., T.O., Y.K., T.D.T., T.N., M.K., and M.Y. approved final version of manuscript.

## FUNDING INFORMATION

This work was funded by Ministry of Education, Culture, Sports, Science and Technology Grants‐in‐Aid JP22K08113 (to M. Y.).

## CONFLICT OF INTEREST STATEMENT

No conflicts of interest, financial or otherwise, are declared by the authors.

## Data Availability

The datasets generated during and/or analyzed during the current study are available from the corresponding author on reasonable request.
